# Competitive Dynamics during Resource-Driven Neurite Outgrowth

**DOI:** 10.1371/journal.pone.0086741

**Published:** 2014-02-03

**Authors:** J. J. Johannes Hjorth, Jaap van Pelt, Huibert D. Mansvelder, Arjen van Ooyen

**Affiliations:** Department of Integrative Neurophysiology, Center for Neurogenomics and Cognitive Research, VU University Amsterdam, Amsterdam, The Netherlands; Stanford University School of Medicine, United States of America

## Abstract

Neurons form networks by growing out neurites that synaptically connect to other neurons. During this process, neurites develop complex branched trees. Interestingly, the outgrowth of neurite branches is often accompanied by the simultaneous withdrawal of other branches belonging to the same tree. This apparent competitive outgrowth between branches of the same neuron is relevant for the formation of synaptic connectivity, but the underlying mechanisms are unknown. An essential component of neurites is the cytoskeleton of microtubules, long polymers of tubulin dimers running throughout the entire neurite. To investigate whether competition between neurites can emerge from the dynamics of a resource such as tubulin, we developed a multi-compartmental model of neurite growth. In the model, tubulin is produced in the soma and transported by diffusion and active transport to the growth cones at the tip of the neurites, where it is assembled into microtubules to elongate the neurite. Just as in experimental studies, we find that the outgrowth of a neurite branch can lead to the simultaneous retraction of its neighboring branches. We show that these competitive interactions occur in simple neurite morphologies as well as in complex neurite arborizations and that in developing neurons competition for a growth resource such as tubulin can account for the differential outgrowth of neurite branches. The model predicts that competition between neurite branches decreases with path distance between growth cones, increases with path distance from growth cone to soma, and decreases with a higher rate of active transport. Together, our results suggest that competition between outgrowing neurites can already emerge from relatively simple and basic dynamics of a growth resource. Our findings point to the need to test the model predictions and to determine, by monitoring tubulin concentrations in outgrowing neurons, whether tubulin is the resource for which neurites compete.

## Introduction

During development, neurons become assembled into functional networks by growing out axons and dendrites (collectively called neurites) that connect synaptically to other neurons. The outgrowth of neurons is mediated by the dynamic behavior of growth cones, specialized structures at the tip of outgrowing neurites. Growth cone migration elongates or retracts the trailing neurite, whereas growth cone splitting creates two daughter branches. Through these growth cone actions, neurons gradually develop their characteristic, highly branched axonal and dendritic trees.

An important but unexplained experimental observation is that elongation of neurite branches is often accompanied by simultaneous retraction of other branches belonging to the same neuritic tree. For example, local calcium influx into an axonal branch [1] or local depolarization of a branch [2] induces rapid outgrowth of the stimulated branch, while at the same time a neighboring branch belonging to the same axon starts retracting. Conversely, cessation of outgrowth in one neurite branch, e.g., as a result of encountering a postsynaptic target neuron, often triggers the outgrowth of its sibling neurite branches [3].

This coordination of neurite outgrowth, occurring both during development and in the restructuring of connectivity during adulthood, is highly relevant for the development and rewiring of synaptic connections [4]–[6]. In the callosal pathway, competitive outgrowth among different neurite branches of the same neuron permits one axon branch to stall or retract while another branch of the same axon extends toward targets [7]. Similarly, depolarization of axonal branches of sympathetic neurons induces outgrowth towards postsynaptic targets at the expense of other branches of the same neuron, which stall or regress [2]. This regulation of neurite outgrowth affects, in an activity-dependent way, the pattern of synaptic connections that will be established. Likewise, local changes in branch outgrowth induced by trophic factors or by chemical or physical cues in the extracellular environment [8] may influence the outgrowth of all the neuron's axonal branches and hence the pattern of synaptic connectivity that will develop.

Competitive interactions among neurites of the same neuron are little studied and the underlying mechanisms are unknown. None of the existing biophysical models of neurite outgrowth [9]–[13] account for competition and the coordinated outgrowth of neurite branches. A small preliminary simulation study [14], using a very simple model, suggested that coordinated outgrowth might emerge from competition for cytoskeletal building blocks produced in the soma and transported to the growth cones, but this mechanism has never been rigorously investigated. In the present computational study, we investigate this competition hypothesis more thoroughly and in a more detailed model, examining neurite outgrowth in complex arborizations, exploring the influence of transport rates and morphology on competition, and testing whether competition can account for experimental data.

Since microtubule polymers constitute the main cytoskeletal structure in neurites, we chose tubulin as the principal resource that neurites need in order to grow out. We constructed full compartmental models of neuritic trees in which neurite outgrowth is governed by tubulin dynamics. In the models, tubulin dimers are produced in the soma and transported by diffusion and active transport to the growth cones. In the growth cones, the tubulin concentration, together with the rate constants of tubulin assembly/disassembly into microtubules, determines the rate of neurite elongation. The model does not include any other processes involved in neurite outgrowth, such as tubulin polymer transport [15]–[17], microtubule sliding [18], actin dynamics [19]–[21], or transport of membrane vesicles [22] and mitochondria [23]. We deliberately simplified the processes underlying neurite outgrowth in order to investigate what behavior could emerge from basic resource dynamics alone.

We address the following questions with our models: (1) Can the apparent coordination of neurite outgrowth arise from competition for tubulin? When one neurite branch is stimulated to grow out, will the neighboring branches retract (as seen in [1])? (2) How is the retraction of neighboring branches modulated by the rate of diffusion, rate of active transport, path distance (i.e., distance along the neuritic tree) to the stimulated branch, and path distance to the soma? (3) During normal development, neurons operate in an inhomogeneous environment, where some neurite branches grow out while others belonging to the same neuron retract (as observed in [3]). To what extent is competition for tubulin able to predict the growth of one branch on the basis of the growth of the other branches?

The paper is organized as follows. In the Methods section, we present the compartmental model and the equations governing tubulin dynamics and neurite outgrowth, as well as the parameter values that were used in the simulations. In the Results section, we subsequently address competition in a simple branching tree (mimicking the setup of [1]), competition in the complex neuritic tree of a complete neuron, and the power of the model to account for neurite outgrowth and retraction in an outgrowing neuron in culture [3].

In summary, we find that competition between outgrowing branches can emerge from basic dynamics of a growth resource such as tubulin and that competition for such a resource may account for experimental findings on outgrowing axonal arborizations [1], [3]. Furthermore, the model predicts how competition between neurite branches changes with path distance between growth cones, path distance to the cell body, and the diffusion constant and rate of active transport of the growth resource.

## Model and Methods

**Figure 1:**
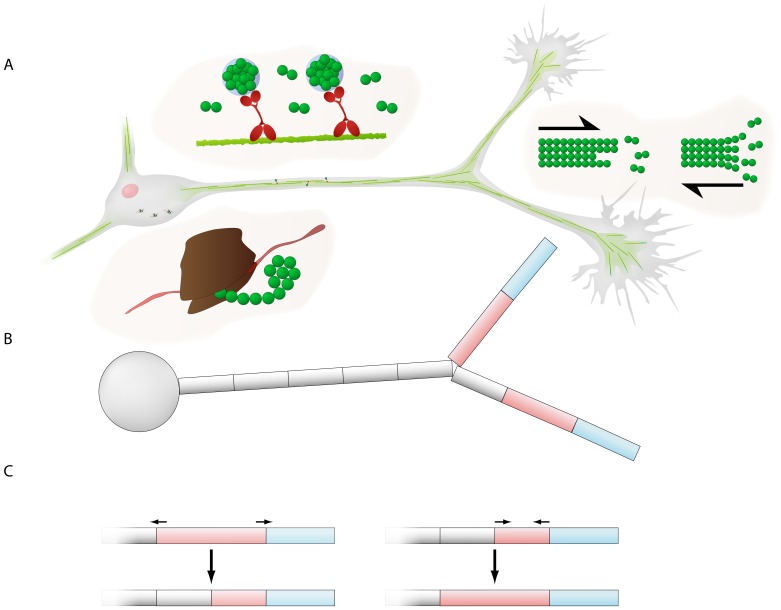
Illustration of the neurite outgrowth model. (A) Tubulin dynamics in the model. Tubulin molecules (green spheres) are produced in the soma, in biological neurons via translation of mRNA on ribosomes (the brown structure). Tubulin is then transported by diffusion and active transport; in biological neurons, the microtubule bundles (the light green fibers) act as railway tracks on which the tubulin molecules are bound via motor proteins (the red molecules). Tubulin is transported to the growth cones at the tip of the neurites. At the growth cone, tubulin is integrated or polymerized into the microtubule cytoskeleton (long polymers of tubulin dimers; the green fibers), which elongates the neurite. When the microtubule depolymerizes, the neurite retracts and tubulin becomes free again. (B) The neuron is divided into multiple compartments. The soma is represented by a single compartment; it connects to a number of neurites consisting of a series of connected compartments. The compartment at the tip of the neurite represents the growth cone (blue). To minimize artificial fluctuations in tubulin concentration, the growth cone is moved forward during growth but its size remains constant, while instead the second compartment (pink) is elongated. (C) The elongating compartment dynamically splits if it becomes too large; likewise, if a shrinking compartment becomes too small, it merges with its proximal (parent) compartment.

To investigate whether competition between sibling neurite branches can emerge from the dynamics of a growth resource such as tubulin, we developed a multi-compartmental model of neurite growth. We assumed that neurite outgrowth is mainly governed by the assembly of microtubules, long polymers of tubulin dimers present throughout the whole neurite (see Fig. 1A). Indeed, treating neurons with toxins that block tubulin polymerization also inhibits neurite elongation [24]. The microtubule cytoskeleton is a necessary component of neurites, functioning both as a stabilizing skeleton and as a railway for active transport. Tubulin is synthesized in the soma, and then transported by diffusion and active transport to the growth cone, where it is polymerized into microtubules [25]–[27].

The model consists of a soma represented by a single compartment that connects to a number of neurites (Fig. 1B). The neurites are divided into a series of connected compartments, with the compartment at the tip of a neurite representing the growth cone. The growth of a neurite is dependent on the local tubulin concentration in the growth cone. However, for numerical reasons, the growth cone is moved during growth but its size remains constant, while instead the second compartment, directly proximal to the growth cone compartment, is elongated. When the second compartment exceeds a certain maximum length (2.5 µm), it is split into two, and when it shrinks below the minimum allowed length (0.5 µm), it is merged with its proximal (parent) compartment (Fig. 1C). By adopting this scheme and not directly changing the size of the growth cone compartment, we minimize artificial fluctuations in volume and concentration [28].

The change in tubulin quantity in the compartments is given by
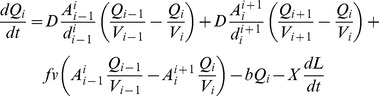
where *Q_i_* is the quantity of tubulin in the *i* ^th^ compartment, *A_i_^i+1^* and *d_i_^i+1^* are the cross-sectional area and distance between the centres of the compartments *i* and *i* +1, respectively. *V_i_* is the volume of compartment *i*. Parameter *f* is the fraction of tubulin present in the compartment that is bound to the active transport system; this fraction is transported with speed *v* by the active transport system. Parameter *D* is the diffusion constant of tubulin, and *b* is the tubulin decay. The last term in the equation accounts for the consumption of tubulin at the growth cone during growth, and is only present in the growth cone compartment. *L* is the length of the neurite, and *X* is the tubulin quantity per unit length. Since experimental findings have shown that the majority of tubulin is synthesized in the soma, with less than 1% synthesized in the axon [26], production of tubulin in the model occurs only in the soma. Also, there appears to be regulatory mechanisms controlling the tubulin concentration [29], and in the model the tubulin concentration in the soma is therefore fixed at a constant value *Q*_0_/*V*_0_, where *V*_0_ is the soma volume.

The change in neurite length is determined by the concentration of tubulin in the growth cone compartment:
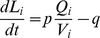
where *p* is the polymerization rate and *q* is the depolymerization rate of microtubules. Note that depolymerization is independent of tubulin concentration because it entails the disassembly of tubulin from existing microtubule bundles.

The model parameters are given in Table 1. The polymerization and depolymerization rates were chosen so that there was no growth at 5 µM [30] and a growth of 0.033 mm/h at 10 µM [31]. With 1640 subunits per µm [32] and around 15 microtubules in a mammalian axon [33], the tubulin quantity per unit length, *X*, is estimated at 4*10^−14^ mol/m. Different values for the tubulin diffusion constant have been reported in the literature: 4.54*10^−13^ m^2^/s [34], 4.3*10^−11^ m^2^/s [35] under the assumption of 1 mM of free Mg^2+^ [36], and 8.59*10^−12^ m^2^/s in the giant squid axon [37]. Here a default value of 10^−11^ m^2^/s was used. The degradation constant for tubulin was taken as 5.67*10^−7^ s^−1^ [38], [39]. For the active transport rate of tubulin we used 440 nm/s, well within the range of experimental values [37], [40], and 0.6% of tubulin was assumed to be bound to the transport network. In all simulations the default values of the parameters were used (Table 1) unless otherwise stated. The somatic tubulin concentration was fixed at 5.5 µM except in the simulations testing the predictive power of the model, where the optimization procedure yielded a value of 17 µM. Both values lie well within the range of values reported experimentally [30], [41], [42]. In order to determine how competition depended on the rates of diffusion and active transport, we investigated a wide range of diffusion constants and active transport rates.

**Table 1:** Default and optimized values of the model parameters.

Parameter	Default value	Optimized value	Unit
*D*	10^−11^	2.15*10^−11^	m^2^/s
*f*	6*10^−3^		
*v*	440*10^−9^	0	m/s
*b*	5.67*10^−7^	5.67*10^−5^	s^−1^
*X*	4*10^−14^		mol/m
*p*	1.83*10^−6^		m/(s mM)
*q*	9.17*10^−9^		m/s
*Q*_0_/*V*_0_	5.5	17	µM

The model was written in Python, and the source code of the model is freely available online at the ModelDB database. Matlab was used for the analysis of the model.

We subsequently address competition in a simple branching tree, competition in a complex branching tree, and the power of the model to account for the outgrowth dynamics of a developing neuron in culture [3]. For the complex branching tree, we used the morphology of two reconstructed rat hippocampal CA1 pyramidal neurons (reconstructed and provided by Martine Groen, Department of Integrative Neurophysiology, VU University Amsterdam). The outgrowth dynamics of a developing neuron was obtained from a time-lapse movie taken of a cerebellar neuron in culture during early development (movie made by Ger Ramakers, Netherlands Institute for Brain Research, Amsterdam). The same movie was also used in [3] for different purposes. Here, we reanalyzed the movie and manually tracked the positions of all growth cones in each frame.

## Results

To investigate whether the apparent competition between growing neurites can emerge from the dynamics of a growth resource such as tubulin, we constructed a multi-compartmental model of a neuron in which it is assumed that the rate of neurite outgrowth depends on the concentration of free tubulin, the building block of the polymerized microtubule cytoskeleton, and the polymerization and depolymerization rates of microtubules. The tubulin is produced in the soma, and transported by diffusion and active transport into the neurites. In the growth cone, a specialized structure at the end of growing neurites, the tubulin concentration, together with the rate constants of tubulin assembly/disassembly into microtubules, determines the rate of neurite elongation.

### Competition in a simple branching tree

**Figure 2:**
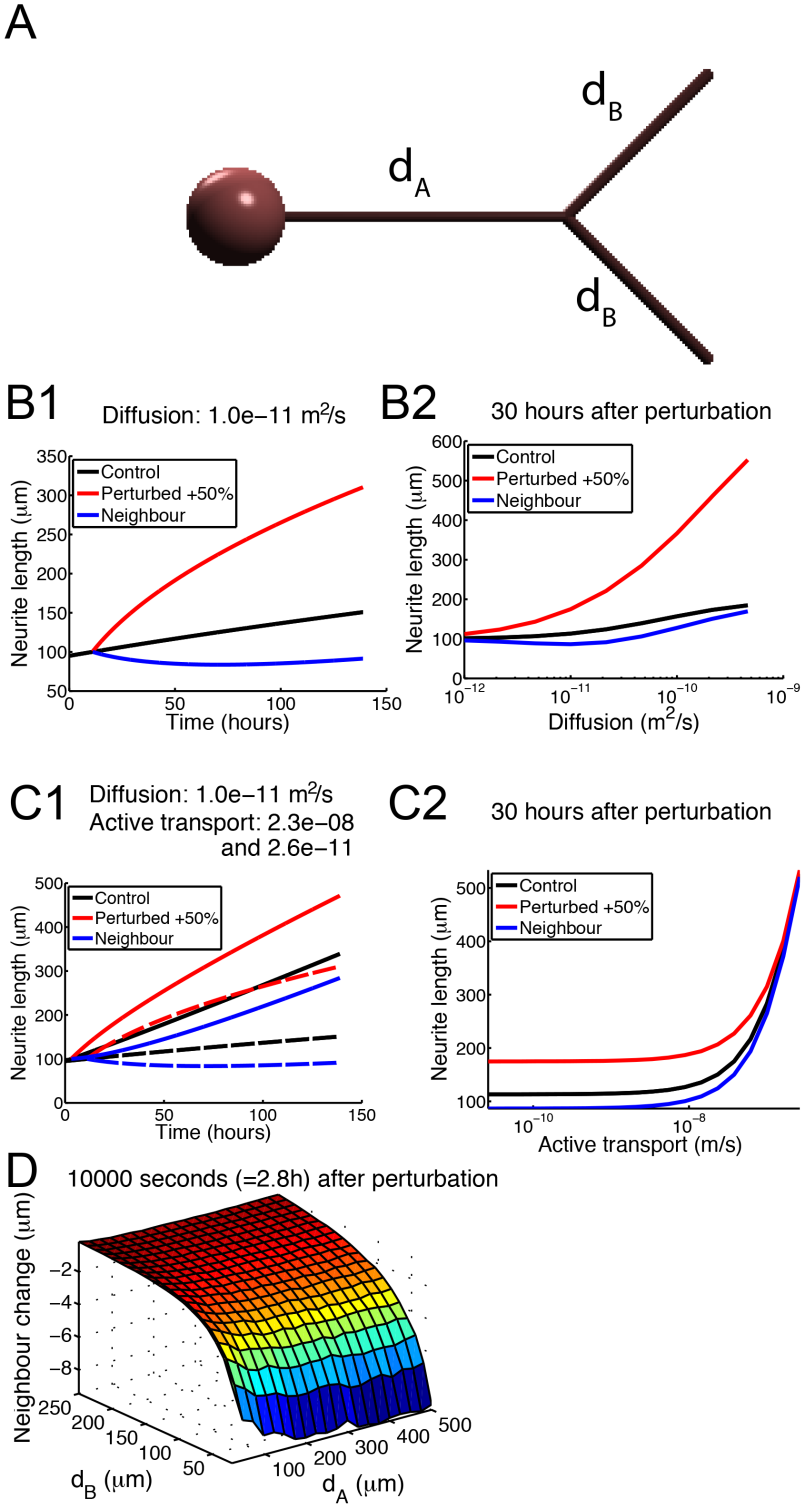
Competition between neurite branches in a simple branching structure. (A) 3D rendering of the model neuron with a soma and a single neurite that splits into two daughter braches at path distance d_A_ from the soma; the two daughter branches have each an initial length of d_B_. (B1) Branches compete when tubulin is only transported by diffusion. Increasing the polymerization rate in one growth cone by 50% (called perturbation) causes the corresponding branch to grow faster (red), at the expense of the neighboring branch, which retracts (blue). The black line shows the control scenario in which there was no change in polymerization rate and both branches grew out at the same speed. Diffusion constant is 10^−11^ m^2^/s. (B2) Shown are the neurite lengths from the soma to the growth cones 30 hours after the perturbation as a function of the tubulin diffusion constant. (C1) Active transport changes the dynamics between the growth cones, reducing the strength of competition. Solid lines indicate a scenario with a high active transport rate (2.3*10^−8^ m/s), where the neighboring branch does not retract after the perturbation. Dashed lines indicate a situation with a low active transport rate (2.6*10^−11^ m/s), where, as with diffusion only, the neighboring branch retracts. (C2) Increased active transport reduces the difference between the lengths of the two branches. Shown are the neurite lengths from the soma to the growth cones 30 hours after the perturbation as a function of the active transport rate. (D) Distance dependence of tubulin competition, 10000 seconds into the simulation. The competition between the growth cones increases (i.e., larger retraction of the neighboring branch) as the path distance d_A_ between soma and branch point increases. The competition decreases with increasing path distance d_B_ from the branch point to growth cones (and thus increasing separation between the growth cones).

First, a simple morphology is used to investigate whether competition between outgrowing branches can arise from tubulin dynamics, and what the effects are of path distance and active and diffusive transport on the interactions between the branches. The model setup mimics the experiments in [1], in which it was observed that stimulating the growth of one branch (by calcium uncaging) triggered the simultaneous retraction of neighboring branches. The model soma had a single neurite, which branched after a path distance d_A_ from the soma, and each of the two branches had an initial length of d_B_ (Fig. 2A). The tubulin concentration at the soma was fixed at 5.5 µM.

In the control case, the polymerization and depolymerization rates at the two growth cones are the same, and the neurite branches grow out at identical speed (Fig. 2B1, black line). When after 10 hours of outgrowth the polymerization rate in one of the growth cones (red line) is increased by 50%, the growth speed of that branch (“the modified branch”) increases at the expense of the neighboring branch (blue line), which starts retracting. Thus, as in the experiments [1], stimulating the growth of one branch induces the retraction of the neighboring branch. The growth cone with the higher polymerization grows faster, uses up more tubulin and has a lower tubulin concentration. As a result, the modified branch has a steeper tubulin gradient and therefore a higher diffusive influx of tubulin, at the expense of the other branch. However, with increased path distance between the growth cone and the branch point, the steepness of the tubulin gradient between them decreases, reducing the tubulin flux into the modified branch and causing the influence of the modified branch on its neighbor to diminish, to the point where the neighboring branch can start growing out again (around 75 hours; see Fig. 2B1).

Fig. 2B2 shows the neurite lengths from the soma to the growth cones 30 hours after the perturbation as a function of the tubulin diffusion constant. The graph shows that, initially, increased mobility of tubulin causes a larger retraction of the neighboring branch. However, at higher rates of diffusion, the retraction becomes smaller, as both branches can now receive enough tubulin from the soma to grow out. Adding active transport to the model causes a fraction of tubulin to be actively moved into each branch, reducing the competition between the branches (Fig. 2C1). Dashed lines in Fig. 2C1 indicate the scenario where active transport represents a small share of the total transport of tubulin. As can be seen, low active transport also results in retraction of the neighboring branch, similarly to the scenario with only diffusion. The solid lines in Fig. 2C1 indicate growth in a simulation with high active transport. In this case, there is no retraction of the neighboring branch after the perturbation, and both branches keep growing out, although at different speeds. Fig. 2C2 shows the neurite lengths from the soma to the growth cones 30 hours after the perturbation as a function of the rate of active transport. Increased active transport attenuates competition and causes the growth of the two branches to become more similar because tubulin resources are now actively moved to both growth cones.

Finally, the dependence on d_A_ and d_B_ of the growth change in the neighboring branch is shown in Fig. 2D. In general, increased path distance between the source of tubulin (soma) and the sink (growth cone) causes the tubulin gradient to become shallower, which reduces the diffusive flux. For short d_B_ distances, we see with increasing d_A_ an increased competition between the two branches (i.e., a larger retraction in the neighboring branch), because the tubulin influx from the soma into the branches decreases with increasing d_A_, implying that the modified branch instead needs to recruit tubulin from the neighboring branch. However, when the path distance d_B_ is increased, the two growth cones become more isolated from each other, and the diffusive flux from one neighbor to the other becomes smaller, so competition decreases.

### Competition in a complex branching tree

**Figure 3:**
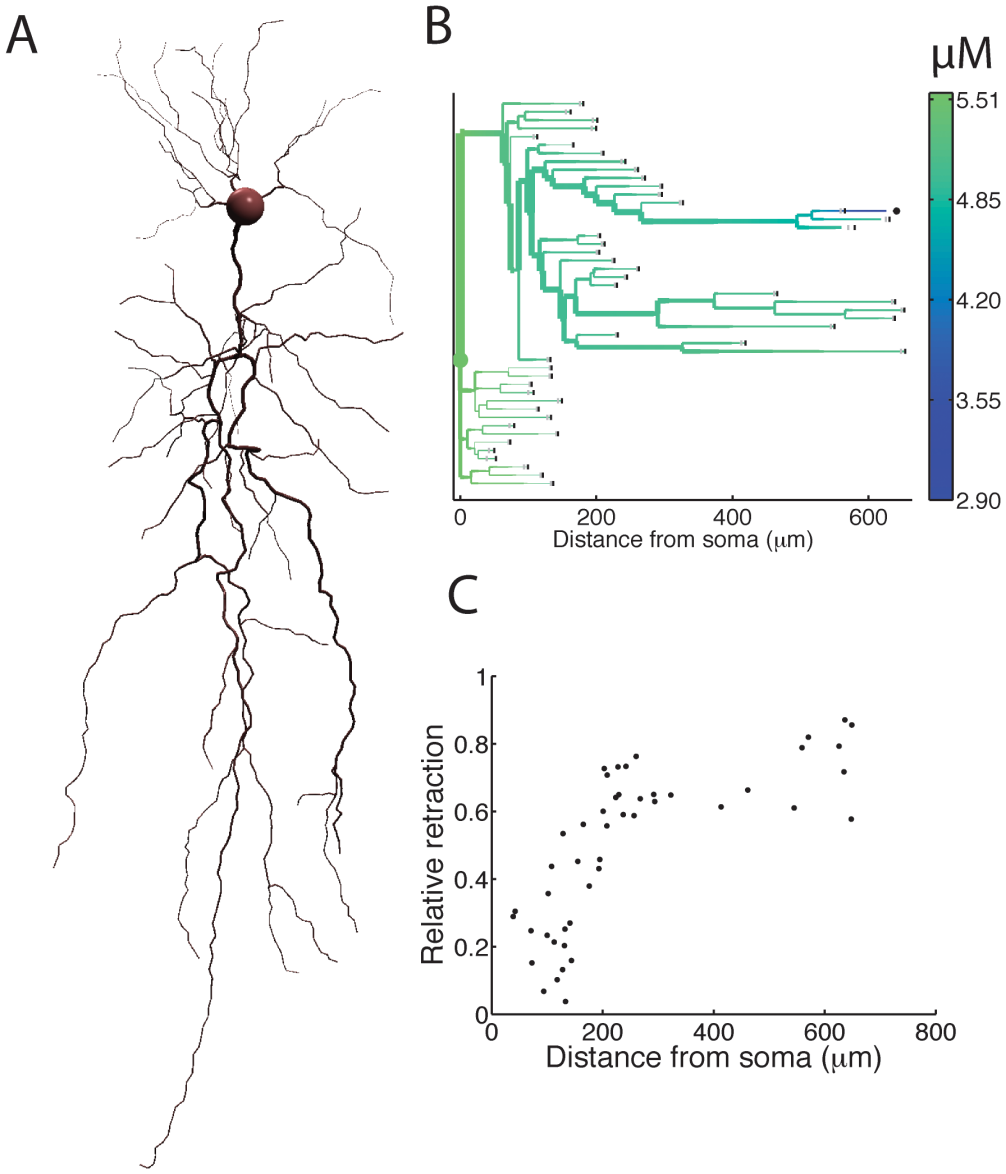
Competition between neurite branches in a complex morphology. (A) Example morphology of a reconstructed pyramidal neuron with apical and basal dendrites. (B) In the control case, starting from the reconstructed morphology, the neuron was allowed to grow out for 10 hours in the model. The simulation was then repeated with the same initial conditions, but with increased polymerization rate for one of the growth cones. The dendritic morphology obtained in this last simulation is represented by a dendrogram, colored according to the tubulin concentration in the branches. The gray vertical lines at the terminal segments indicate the starting morphology, and the black vertical lines show the neurite length after 10 hours in the control case. The black dot marks the growth cone with increased polymerization rate. (C) The competition between branches increases with increasing path distance to the soma. The graph shows the total retraction of all neurites, divided by the growth of the modified growth cone, as a function of path length between the modified growth cone and the soma.

To study competitive interactions in more complex branched structures, we used the morphology of two reconstructed rat hippocampal CA1 pyramidal neurons. Here, we show the results of only one neuron (Fig. 3), but the findings of both neurons were consistent with each other. The reconstructed morphology of the apical and basal dendrites (Fig. 3A) was used as the initial condition. In the control case, the neuron was allowed to grow out for 10 hours with the soma tubulin concentration fixed at 5.5 µM. The simulation was then repeated, but now one of the growth cones had an increased polymerization rate (+100%). In Fig. 3B, the dendritic morphology obtained in the last simulation is represented by a dendrogram, colored according to the tubulin concentration in the branches. The thickness of the branches is proportional to the dendrite diameter in the reconstruction. The gray vertical lines at the terminal segments indicate the starting morphology, and the black vertical lines show the neurite length after 10 hours in the control case. The black dot marks the growth cone with increased polymerization rate. As can be seen in Fig. 3B, the terminal branch with the modified growth cone increased its length at the expense of its most nearby terminal branches, i.e., its sibling terminal branch and its parent's sibling branch, which both retracted. The retraction is larger in the thicker terminal branch. This may indicate that, as with higher diffusion rates (Fig. 2B2), larger branch diameters cause more flux from the neighboring branches into the modified branch and thus stronger competition between the branches. The terminal branches that were more remote from the modified branch showed very little retraction.

We then carried out more simulations with modified polymerization rate, in each simulation selecting another growth cone that had its polymerization rate increased. In each case, the total retraction at all terminal branches divided by the growth of the modified branch was calculated. In Fig. 3C, this total relative retraction is plotted against the path length between the modified growth cone and the soma. The data show that the total relative retraction increases with larger path lengths between modified growth cone and soma. This confirms what we observed in the simplified morphology (see Fig. 2D), namely that the more isolated the neurites were (larger path distance d_A_ from the soma), the stronger the competition was between them.

### Predictive power of the model

**Figure 4:**
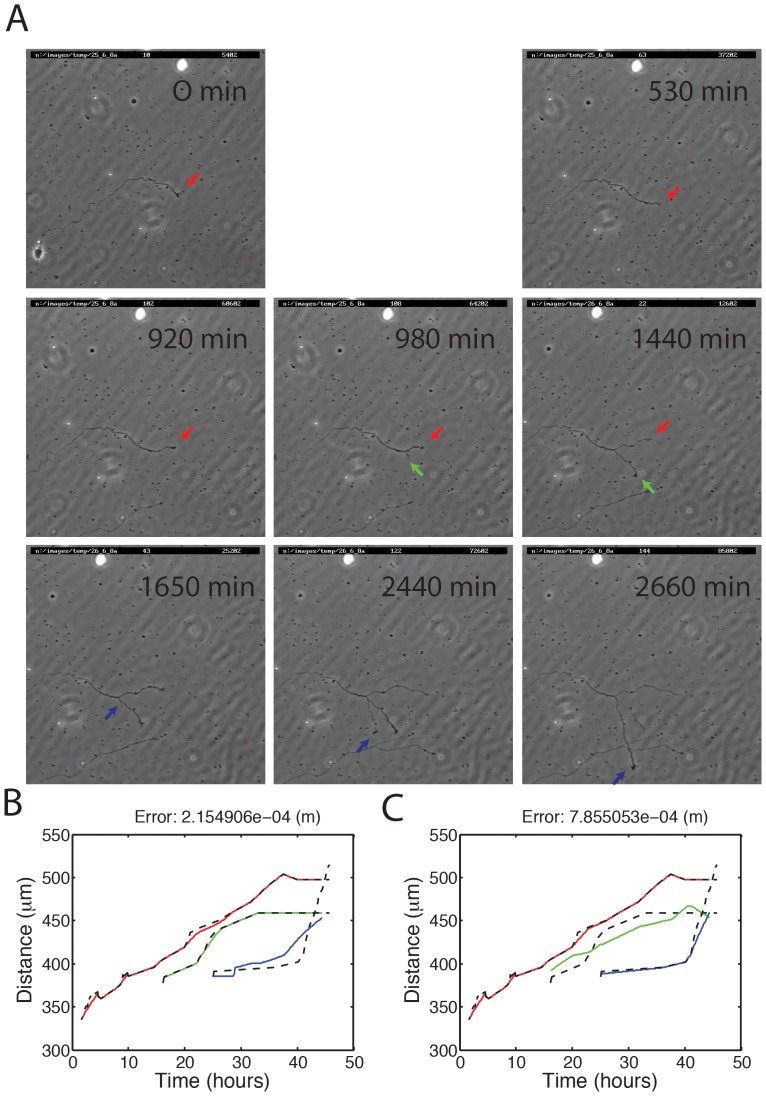
Neurite outgrowth of a developing neuron in tissue culture. (A) Still shots of a time-lapse movie of a developing cerebellar neuron in tissue culture, revealing neurites that are growing out and retracting. The arrows point to the neurites' growth cones; color of arrows corresponds to colors used in panels B and C. Figure taken from [3]. (B) The red and green neurites are forced to grow out as in the experiment (dashed black lines), whereas the blue neurite is fully controlled by the tubulin dynamics of the model. The parameters of the model (diffusion constant, active transport rate, tubulin decay and tubulin soma concentration) were optimized so as to make the blue neurite grow as closely as possible to the experimental data. (C) Using the optimized parameter set from B, the green neurite is now fully governed by the model, whereas the red and blue neurites are forced to grow according to data recorded in the experiment. The errors in B and C are the square root of the summed squared deviation of the free growth cone from the experimentally measured location at each point in time.

To investigate the predictive power of the model, we used as reference a time-lapse movie of an outgrowing neuron in a culture dish, revealing neurites that branch, grow out and retract in a dynamical fashion [3]. A selection of video frames is shown in Fig. 4A. The growth cone movements were manually detected in each frame of the video. At the start of the movie a single neurite (red) grows out. After 980 minutes a second neurite (green) has formed and starts extending in parallel with the first neurite. After 1650 minutes a third neurite appears (blue), initially growing slowly, but then starting to grow out more rapidly at the expense of the other neurites. In tissue culture, the rate of neurite outgrowth may be affected by molecular guidance molecules and other chemical and physical cues in the cell's environment. The model does not explicitly include such cues.

The question we asked was whether the model could predict the behavior of one growth cone given the behavior of the other two growth cones. To this end, two of the neurites in the model were forced to grow out at the same speed as the corresponding neurites in the experiment, while the third neurite was fully controlled by the model. A neurite's growth was forced by changing the neurite's distal coordinate and removing the amount of tubulin in the growth cone that was required for such a length change; however, if that produced a negative tubulin concentration, the neurite was not allowed to grow out. Depending on the growth and retraction of the first two neurites (red and green), the tubulin concentration in the third growth cone (blue) would fluctuate, resulting in varying growth speeds of the neurite. The polymerization and depolymerization rates were fixed at their default values (see Methods). The tubulin concentration in the soma, the active transport rate, diffusion constant and the tubulin decay rate were optimized, but fixed during the course of one simulation, to give as close a match as possible between experiment and model result for the blue growth cone (Fig. 4B). The parameters were optimized by discretizing the parameter space and doing an exhaustive search (whereby the diffusion constant *D* could vary from 1*10^−13^ to 0.5*10^−10^ m^2^/s, the rate of active transport *v* from 0 to 440*10^−7^ m/s, the tubulin decay *b* from 5.67*10^−7^ to 5.67*10^−4^ s^−1^, and the soma concentration *Q*_0_/*V*_0_ from 5.5 to 50 µM). The summed deviation of the free growth cone from the experimentally measured location at each point in time was used as an error measure.

As seen in Fig. 4B, at the start of the simulation the red neurite grows out and follows the experimental trace quite closely; then after 980 minutes the green neurite buds off and starts growing out. The slight discrepancy of the red and green traces with the experimental traces (dashed lines) is due to the model constraint that if a forced growth cone does not have enough tubulin it cannot grow out. By 1650 minutes into the experiment, the blue neurite—the free neurite, whose growth is fully governed by the model—forms but initially grows out very slowly. The fast outgrowing red and green neurites create a steep tubulin gradient and a large diffusive influx and consume most of the tubulin resources, hampering the outgrowth of the blue neurite. When the red and green neurites stall, more resources become available to the blue growth cone, enabling the blue neurite to grow out faster. Although the stalling of the red and green neurites are seen to trigger the faster outgrowth of the blue neurite, as in the experiment, none of the parameter sets we tried were able to completely capture the sudden rapid elongation of the blue neurite.

As a way of verifying the optimized parameter set, the simulation was then repeated, but this time the red and the blue neurites were forced to grow out as in the experimental movie, and the green one was controlled by the model (Fig. 4C). This can be regarded as a weak form of cross-validation: in the previous simulation we searched for a parameter set that optimized the outgrowth of the blue neurite, and in this simulation we tested, using the same parameter set, the readout of the green neurite. As expected, the blue neurite is now much closer to the experimental trace (dashed line) because it is being forced to match it; however, there is not quite enough tubulin available to precisely match the rapid elongation commencing around 40 hours. The green neurite—the free neurite controlled by the model—follows the experimental trace approximately. As a result of the competitive interactions between the neurites, around 32 hours the green neurite slightly reduces its growth speed when the red neurite increases its growth speed; the reduction in growth speed is not as large as in the experiment, which shows that the green neurite then stops growing out. Around 40 hours, the green neurite starts retracting when the blue neurite exhibits a growth spurt.

Overall, the model is able to capture at least the qualitative behavior of the experimental neuron. In tissue culture, external chemical and physical cues in the neuron's environment, which are not implemented in the model, are also likely to influence outgrowth. With that in mind, the model performs reasonably well in accounting for the growth dynamics.

## Discussion

During neuron outgrowth, the elongation of neurite branches is often accompanied by the simultaneous retraction of other branches in the same neuron [1]–[3]. These apparently competitive interactions are important in the formation of synaptic connections [2], [4]–[7], but the biological processes underlying this coordination of neurite outgrowth are poorly known. Using a multi-compartmental model of neurites, we have shown here that competition between outgrowing neurite branches can already emerge from relatively simple dynamics of a growth resource such as tubulin. Tubulin is the building block of the microtubule cytoskeleton, a key structure in neurites that provides stability and rigidity.

In the model, just as in experimental studies [1], stimulating the outgrowth of a neurite branch can lead to the simultaneous retraction of sibling branches. The model predicts that the amount of retraction decreases with increasing path distance between the branches' growth cones, increases with increasing path distance between growth cone and soma, decreases with increasing rate of active transport, and initially increases with increasing diffusion constant. We confirmed that competitive interactions between outgrowing branches can occur not only in simple morphologies but also in the complex dendritic morphology of pyramidal neurons. Also in these complex morphologies, we found that the more isolated the growth cones are from the soma, the stronger they compete with each other. Furthermore, we showed that, in a developing neuron in tissue culture [3], competition for a growth resource such as tubulin may be able to predict, at least qualitatively, the growth of one neurite branch on the basis of the growth of the other branches.

As mentioned, the model shows that stimulating the outgrowth of a neurite branch can lead to the simultaneous retraction of neighboring branches. In the model, the length increase of the growing branch was always larger than the length decrease of the retracting branch. In the experiments [1], however, the retraction was sometimes larger than the elongation. This difference between model and experiment could be the result of internal regulation of growth not captured by our simple model. For example, the model does not include any regulation of active transport.

The model could not fully account for the initial suppression of the outgrowth of the blue neurite as seen in the experiment (Fig. 4B). This could be because, in addition to competitive interactions with the other two neurites, there were external cues in the tissue culture hindering the outgrowth of the blue neurite. Alternatively, the initial suppression of the blue neurite in the experimental data might be due to actin dynamics in the neurite (not included in the model). Actin structures in the growth cone and retrograde flow of actin filaments are known to regulate microtubule dynamics and neurite outgrowth [19], [20]. Besides regulating growth rate, actin in the growth cone is also critically involved in neurite guidance and steering [21]. After around 38 hours, the growth of the blue neurite is slower in the model than in the experiment (Fig. 4B). The faster outgrowth in the experiment could be due to other mechanisms of tubulin transport besides diffusion and active transport of tubulin dimers, such as microtubule polymer transport [15]–[17] and microtubule sliding [18]. Sliding of microtubules against each other might also have a direct role in competition between neurite branches, especially during early neuronal development [18]. Microtubule sliding provides mechanical forces for neurite protrusion over long distances, and microtubules could slide between one neurite tip and another.

Obviously, neurite outgrowth is more complex than implemented in the model, and involves in addition to tubulin dynamics, actin dynamics [19]–[21], transport of vesicles supplying membrane for neurite extension [22], and transport of mitochondria providing energy [23]. In the model, we deliberately simplified the process of neurite outgrowth in order to investigate what behavior could already emerge from basic tubulin dynamics alone. Tubulin was assumed to be the rate-limiting resource for neurite outgrowth. Although microtubule polymers are indeed a main component of the neurite's cytoskeleton, with tubulin consequently being a necessary resource for neurites to grow out, we would obtain very similar results if any other resource was rate limiting for outgrowth, so long as this resource is produced in the soma and transported by diffusion and active transport to the growth cones and there consumed to elongate the neurite. Thus, from our model results we cannot infer that tubulin is necessarily involved in competition, only that a rate-limiting resource with the same type of simple dynamics as implemented here for tubulin is capable of producing competitive interactions between outgrowing neurites.

Our model predictions—that competition between neurite branches decreases with path distance between the growth cones, increases with path distance to the cell body, and is mitigated by a greater share of active transport in the total transport of tubulin—are amenable to experimental testing. To test the dependence on path distance to the cell body, the experiments in [1], in which the growth of one branch was stimulated by local calcium uncaging and the growth of the other branches monitored, can be repeated by systematically selecting branches for stimulation that are at various path distances from the soma. To test the dependence on path distance between growth cones, the growth response of the other branches can be plotted as a function of path distance to the stimulated branch. The rate of active transport in neurites may also be altered experimentally [43] in order to test whether a lower rate leads to enhanced competition, as the model predicts.

The competitive interactions between neurite branches that we observed in the model depend on tubulin being transported, at least partially, by diffusion. If indeed tubulin is the rate-limiting resource for neurite outgrowth, the model therefore predicts that there should be a spatial gradient of tubulin from high levels in the soma to lower levels in the growth cone. Furthermore, the gradient in the fastest outgrowing neurite branch should initially be steeper, as more tubulin is consumed in the growth cone of this branch than in the growth cones of the other branches. Because of the steeper gradient, there will be a larger diffusive influx into the fastest growing branch as compared to the other branches. These predictions can be tested experimentally by monitoring the concentration gradient of free tubulin in outgrowing neurites.

To further test whether competition for a growth resource such as tubulin is able to predict the growth of one branch on the basis of the growth of the other branches, it would be desirable to acquire more time-lapse movies of growing neurites, and perform analyses similar to those in Fig. 4. Analysing more movies would have made our conclusions stronger, but these movies are currently not available. Our results highlight the importance of such detailed movies for investigating the mechanisms underlying the complex dynamics of neurite outgrowth.

Only a few biophysical models of neurite outgrowth exists [13]. Van Ooyen et al. [14], in a small pilot experiment, studied neurite outgrowth based on tubulin dynamics in a highly simplified model consisting of only three compartments, a soma compartment and two growth cone compartments. They found that the fastest growing neurite branch could prevent the outgrowth of the other branch. However, they did not study retraction of neurites, or the influence of diffusion constant, active transport rate and path distance from the soma and between growth cones on the competitive interactions between neurites. In a more extensive compartmental model [9], neurite outgrowth was modulated by microtubule-associated proteins (MAPs), with phosphorylated MAP2 favoring branching and dephosphorylated MAP2 favoring elongation. The authors showed that depending on the relative rates of calcium-dependent phosphorylation and dephosphorylation, a variety of characteristic dendritic trees was produced, but they did not investigate competitive interactions between neurite branches. Another biophysical model of neurite outgrowth was based on membrane expansion by exocytosis of vesicles transported inside the cell body and neurite [12], but this model was also not concerned with competition. Likewise, models that focus on the effects of external influences on neurite outgrowth, such as adhesion between neurite and substrate [11] or repulsive interactions between neurites [10], did not examine possible competitive effects between neurites. Some form of competition was studied in a model of axon-dendrite differentiation based on shootin 1 [44]. It was shown that anterograde transport and retrograde diffusion of shootin 1, together with shootin 1-dependent neurite outgrowth, can cause one neurite to outgrow its siblings and become the axon.

In contrast to biophysical models, phenomenological models of neurite outgrowth do not directly implement the underlying biological mechanisms responsible for neurite elongation and branching. In the stochastic phenomenological model of Van Pelt et al. [45]–[47], each growth cone in the growing tree has a certain probability to branch and elongate. Interestingly, to be able to accurately produce the morphology of a wide range of neuron types, the model needs a competition factor describing how the growth cone's actions depend on the momentary total number of growth cones in the tree. This factor may reflect competition between growth cones for resources such as tubulin [31].

In conclusion, our results suggest that competition between outgrowing neurites can already arise from basic dynamics of a growth resource such as tubulin and that competition for such a resource may partly underlie the experimentally observed differential outgrowth and retraction of neurite branches of the same neuron.
